# Evidence of weaker phenotypic plasticity by prey to novel cues from non‐native predators

**DOI:** 10.1002/ece3.2271

**Published:** 2016-07-02

**Authors:** Johan Hollander, Paul E. Bourdeau

**Affiliations:** ^1^ Department of Biology, Aquatic EcologyLund UniversityEcology BuildingSE‐223 62LundSweden; ^2^ Department of Biological SciencesHumboldt State University1 Harpst St.ArcataCalifornia95521

**Keywords:** Coevolution, inducible defensive traits, meta‐analysis, naive interactions

## Abstract

A central question in evolutionary biology is how coevolutionary history between predator and prey influences their interactions. Contemporary global change and range expansion of exotic organisms impose a great challenge for prey species, which are increasingly exposed to invading non‐native predators, with which they share no evolutionary history. Here, we complete a comprehensive survey of empirical studies of coevolved and naive predator−prey interactions to assess whether a shared evolutionary history with predators influences the magnitude of predator‐induced defenses mounted by prey. Using marine bivalves and gastropods as model prey, we found that coevolved prey and predator‐naive prey showed large discrepancies in magnitude of predator‐induced phenotypic plasticity. Although naive prey, predominantly among bivalve species, did exhibit some level of plasticity – prey exposed to native predators showed significantly larger amounts of phenotypic plasticity. We discuss these results and the implications they may have for native communities and ecosystems.

## Introduction

Phenotypic plasticity is a developmental strategy where an individual's genotype has the ability to interact with its environment and produce different phenotypes. The resulting phenotypic flexibility may increase an organism's fitness in a heterogeneous environment and it is a common attribute across many taxa (Schlichting 1986; Appleton and Palmer 1988; Whitman and Ananthakrishnan 2008; Hollander and Butlin 2010).

Phenotypic plasticity is not considered likely to evolve if accessible cues are not reliable predictors of environmental change to the organism (Moran 1992; Tufto 2000). Indeed, in order for adaptive phenotypic plasticity to evolve, predictable environmental cues, such as seasonal or systematic environmental change, or chemical signals indicating the presence of a coevolved predator, are essential. Coevolution is the result of a shared evolutionary history between two or more species that reciprocally affect each other's evolution (Ehrlich and Raven 1964; Dawkins and Krebs 1979; Brodie and Brodie 1999). Thus, the chemical signature of a coevolved predator is likely to represent a reliable cue indicating risk to a prey organism. However, since rapid globalization of the world has increased species mobility and led to a growing number of alien introductions of both animals and plants, including many predatory species, prey species are increasingly being exposed to cues from predators with which they share no evolutionary history (so‐called naive interactions). Native prey species that are naive to cues and unable to alter their phenotype in response to novel predators, or native prey that are less flexible in their ability to alter their phenotype in response to novel predators will accordingly experience reduced fitness and a potentially larger extinction risk when exposed to non‐native invading, or range‐expanding predators.

In accordance with theory, which predicts that adaptive phenotypic plasticity will fail to evolve without reliable cues (Moran 1992; Tufto 2000), there are a number of examples where naive prey fail to produce phenotypically plastic defensive traits (i.e., inducible defenses) when exposed to novel predators (Edgell and Rochette 2007; Edgell and Neufeld 2008). However, in other cases, naive prey have also been shown to respond plastically to novel predators (Freeman and Byers 2006; Rawson et al. 2007; Bourdeau et al. 2013; Freeman et al. 2014; Hooks and Padilla 2014). Thus, the general importance of evolutionary history/novelty in mediating plastic prey responses to predators is still not well understood; accordingly, a more comprehensive approach is called for to improve our understanding concerning the evolution of adaptive phenotypic plasticity in novel predator−prey interactions.

Marine molluscs such as bivalves and gastropods have a long history in the study of inducible defensive traits (Appleton and Palmer 1988; Leonard et al. 1999; Trussell and Nicklin 2002; Edgell and Neufeld 2008; Bourdeau 2009, 2010a; Freeman and Hamer 2009; Hollander and Butlin 2010). These taxonomic groups have served as a model owing to a number of beneficial characteristics, such as their accessibility on rocky and sandy shore habitats, well‐defined defensive traits that can easily be quantified, and amenability to rearing in laboratory environments. For that reason, there are numerous studies available in the published literature. Here, we provide a test where we synthesize previously published data, with the purpose of examining whether prey species that are exposed to non‐native predators (those without a shared evolutionary history), whether prey show reduced phenotypic plasticity in response to novel predators (those without a shared evolutionary history) compared to prey that are exposed to native predators (with which they share an evolutionary history). A benefit of this study system is the fact that several of the species included in the present analysis are represented in each of the two contrasting groups: (1) naive native prey exposed to non‐native predators; and (2) experienced native prey exposed to native predators.

## Methods

We used ISI *Web of Science* (*WoS*) database and search engine to sample multiple independent studies published in the peer‐reviewed scientific literature. We used the following search string to identify relevant papers by: Topic = (snail* OR gastropod*) AND (plastic* OR phenotyp* plastic* OR induce*) and (bivalve* or mussel* or clam* or oyster*) AND (plastic* OR phenotyp* plastic* OR induce*). We only searched English language publications.

We inspected the collected papers to determine whether they focused on examining plastic responses of bivalve and gastropod phenotype (e.g., behavior, morphology, life‐history) to predators. Studies that did not meet our criteria were omitted from the data set. A number of studies were rejected during this process because they were not related to the topic of bivalve or gastropod phenotypic plasticity or they focused on the plasticity of predators rather than on the plasticity of bivalve or gastropod prey. The publication records in our data set covered from 1987 to 2014 (Table 1) and identified 274 studies. In addition to our literature exclusion process, we also searched the references in the collected papers for supplementary literature that was missed through keyword search. The data set has been submitted to DOI: doi:10.5061/dryad.b06f9.

**Table 1:** The studies used in the meta‐analysis. The table is sorted by author

Author	Year	Journal	Class	Species	Predator	Predator or prey: invasive or native
Behrens‐Yamada	1998	*J Exp Mar Biol Ecol*	Gastropod	*Littorina sitkana*	*Cancer productus* (crab)	Native
Bibby et al.	2007	*Biol Lett*	Gastropod	*Littorina littorea*	*Carcinus maenas* (crab)	Native
Bourdeau	2009	*Ecology*	Gastropod	*Nucella lamellosa*	*C. productus/Pisaster ochraceus* (sea star)	Native
Bourdeau	2010a	*Pro R Soc B*	Gastropod	*Nucella lamellosa*	*Cancer productus*	Native
Bourdeau	2010b	*Oecologia*	Gastropod	*Nucella lamellosa*	*C. productus*	Native
Bourdeau	2011	*Func Ecol*	Gastropod	*N. ostrina/canaliculata/lamellosa*	*Cancer productus*	Native
Bourdeau	2012	*J Anim Ecol*	Gastropod	*Nucella lamellosa*	*Cancer productus*	Native
Brookes & Rochette	2007	*J Evol Biol*	Gastropod	*Littorina obtusata*	*Carcinus maenas*	Invasive
Caro et al.	2008	*Mar Ecol Progr Ser*	Bivalve	*Perumytilus* sp.*/Semimytilus* sp.	Various predators	Native
Cheung et al.	2004	*Marine Biology*	Bivalve	*Perna viridis*	*Thais sp*. (whelk)/*Thalamita* sp. (crab)	Native
Dalziel & Boulding	2005	*J Exp Mar Biol Ecol*	Gastropod	*Littorina subrotundata*	*Hemigrapsus nudus* (crab)	Native
Edgell & Neufeld	2008	*Biol Lett*	Gastropod	*Nucella lamellosa*	*C. maenas/C. productus*	Invasive/Native
Edgell et al.	2009	*Am Nat*	Gastropod	*Littorina obtusata*	*Carcinus maenas*	Invasive
Fässler & Kaiser	2008	*Mar Ecol Progr Ser*	Bivalve	*Mytilus edulis*	*Carcinus maenas*	Native
Freeman & Byers	2006	*Science*	Bivalve	*Mytilus edulis*	*Hemigrapsus sanguineus*	Invasive
Freeman	2007	*Mar Ecol Progr Ser*	Bivalve	*Mytilus edulis*	Various predators	Invasive/Native
Freeman & Hamer	2009	*J Exp Mar Biol Ecol*	Gastropod	*Nucella lapillus*	*C. maenas/Asterias rubens* (sea star)	Invasive/Native
Freeman et al.	2009	Oikos	Bivalve	*Mytilus edulis/trossulus*	Various predators	Invasive/Native
Freeman et al.	2014	J Exp Mar Biol Ecol	Gastropod	*Nucella lapillus*	*C. maenas/Cancer* spp.	Invasive/Native
Hollander & Butlin	2010	*J Evol Biol*	Gastropod	*Littorina saxatilis*	*Carcinus maenas*	Native
Hollander et al.	2006	*J Evol Biol*	Gastropod	*Littorina saxatilis*	*Carcinus maenas*	Native
Hooks & Padilla	2014	*J Exp Mar Biol Ecol*	Gastropod	*Littorina saxatilis*	*Dyspanopeus sayi/Hemigrapsus sanguineus*	Invasive/Native
Johnson	2014	*Marine Biology*	Bivalve	*Crassostrea virginica*	Various predators	Native
Leonard et al.	1999	*Ecology*	Bivalve	*Mytilus edulis*	*Carcinus maenas*	Invasive
Lord & Whitlatch	2012	*Marine Biology*	Bivalve	*Crassostrea virginica*	*Urosalpinx cinerea* (whelk)	Native
Lowen et al.	2013	*Mar Ecol Progr Ser*	Bivalve	*Mytilus edulis*	Various predators	Native
Nakaoka	2000	*Ecology*	Bivalve	*Mercenaria mercenaria*	*Busycon carica* (whelk)	Native
Neo & Todd	2011	*J Exp Mar Biol Ecol*	Bivalve	*Tridacna squamosa*	*Myomenippe hardwickii* (crab)	Native
Pernet	2007	*Amer Malac Bull*	Gastropod	*Amphissa columbiana*	*Cancer productus*	Native
Reimer & Harms‐Ringdahl	2001	*Marine Biology*	Bivalve	*Mytilus edulis*	*Asterias rubens*	Invasive/Native
Reimer & Tedengren	1996	*Oikos*	Bivalve	*Mytilus edulis*	*Asterias rubens*	Native
Rochette et al.	2007	*Mar Ecol Progr Ser*	Gastropod	*Littorina obtusata*	*Carcinus maenas*	Invasive
Sepulveda et al.	2012	*J Molluscan Studies*	Gastropod	*Acanthina monodon*	*Homalaspis plana* (crab)	Native
Shin et al.	2009	*Mar Fresh Behav Phys*	Bivalve	*Perna viridis*	*Thalamita danae* (crab)	Native
Smith & Jennings	2000	*Marine Biology*	Bivalve	*Mytilus edulis*	*N. lapillus* (whelk)/*C. maenas*	Invasive/Native
Trussell	1996	*Evolution*	Gastropod	*Littorina obtusata*	*Carcinus maenas*	Invasive
Trussell	2000	*Evolution*	Gastropod	*Littorina obtusata*	*Carcinus maenas*	Invasive
Trussell	2002	*Mar Ecol Progr Ser*	Gastropod	*Littorina obtusata*	*Carcinus maenas*	Invasive
Trussell & Smith	2000	*PNAS*	Gastropod	*Littorina obtusata*	*Carcinus maenas*	Invasive
Trussell et al.	2002	*Ecology*	Gastropod	*N. lapillus and L. littorea*	*Carcinus maenas*	Invasive
Whitlow	2010	*Marine Ecology*	Bivalve	*Mya arenaria*	*Carcinus maenas*	Invasive

In order to summarize and quantify large sets of data, meta‐analysis has proved to be a powerful statistical tool. To create a consistent estimate of the quantitative measure of phenotypic plasticity, we calculated a common unit, the effect size, using the same methodology as in Hollander (2008); implementing Hedge's *d* (Gurevitch and Hedges 1993) as an effect size to quantify plasticity for each study. The data set was additionally divided into four different variables in order to assess each variable separately. The four variables were as follows: life‐history (comprising growth rate), behavior, shell morphology, and soft tissue morphology. The soft tissue morphology describes the soft part of the animal, and in bivalves this is often adductor mussel size or ‘meat’. We analyze these variables separately for both taxa together, and in gastropods and bivalves exclusively.

Hedge's *d* uses arithmetic means of the phenotype from the experimental group and correspondingly from the control group's mean phenotype. The equation to calculate Hedge's *d* also requires the standard deviation and the sample size for each study from both the experimental and the control group. Studies are likely to vary in the degree of difference between treatments, which may influence the phenotypic effect observed, but meta‐analysis is robust to such variation(Rosenthal 1991) and it is unlikely to be confounded with the origin of the predator (native vs. non‐native). Moreover, since we assumed a random component of variation among effect sizes (Rosenthal et al. 2000), we used a mixed model. The analyses were conducted in the statistical software MetaWin 2.0 (Rosenthal et al. 2000). Additionally, we used what is termed ‘Reversal marker’ in the software, which enables the direction of the effect between the control and the experimental treatments to always be positive (Gurevitch et al. 1992). In order to test for the robustness of data due to publication bias (i.e., when negative results have a lower publication rate compared to positive results [the “file‐drawer effect”; (Rosenthal 1984)], we examined the data using both a funnel graph (Palmer 1999), and a fail‐safe sample size analysis that calculates the number of studies with zero effect that would be required to reject our stated hypotheses (Rosenthal 1979). However, both the funnel graph and Rosenthal's method suggested a robust data set where publication bias was very unlikely (see Results).

## Results

**Figure 1:**
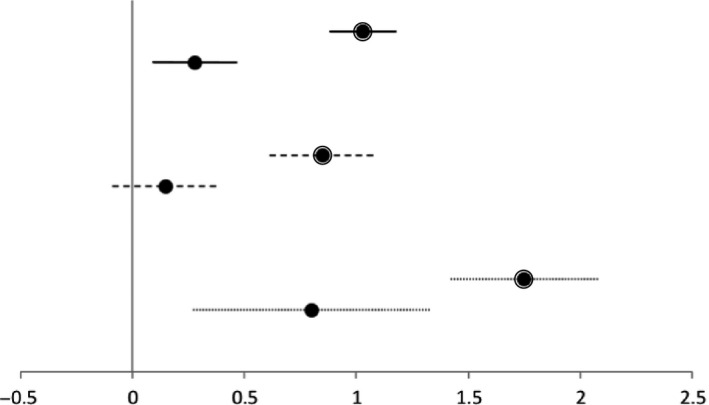
Mean effect sizes (Hedge's *d*) and 95% confidence intervals for the magnitude of phenotypic plasticity between native predator−prey interactions (enclosed circles) and interactions between predators and naive prey. Dotted lines represent bivalve species, dashed lines represent gastropod species, while solid lines illustrate both taxa.

The meta‐analysis compiled 274 studies from 1987 to 2014. Species represented in the data set for the prey exposed to non‐native predators group (*n* = 82 studies) were as follows: *Littorina littorea, Littorina obtusata, Littorina saxatilis, Mya arenaria, Nucella lamellosa, and Nucella lapillus;* and for the native predator−prey interactions group (*n* = 192 studies) were as follows: *Acanthina monodon, Amphissa columbiana, Austrocochlea constricta, Bembicium vittatum, Crassostrea virginica, Littorina keenae, Littorina littorea, Littorina saxatilis, Littorina sitkana, Littorina subrotundata, Mercenaria mercenaria, Mytilus edulis, Mytilus trossulus, Nodilittorina australis, Nucella canaliculata, Nucella lamellosa, Nucella lapillus, Nucella ostrina, Patella barbara, Perna viridis, Perumytilus purpuratus, Semimytilus algosus, Tridacna squamosa, and Turbo coronatus*. The non‐native predator species represented in the data set were as follows: *Asterias rubens, Carcinus maenas,* and *Hemigrapsus sanguineus*. The meta‐analysis including both bivalves and gastropods, found that for the native predator−prey interaction, the group had a mean effect size (*d*_native_ = 1.013; CI: 0.876 to 1.149; df = 191) that was significantly larger than prey experiencing non‐native predators (*d*_non‐native_ = 0.246; CI: 0.060 to 0.432; df = 81), since the two groups CI did not overlap (Fig. 1). We found the same pattern regardless if we analyzed bivalves (*d*_native_ = 1.702; CI: 1.387 to 2.017; df = 70), (*d*_non‐native_ = 0.535; CI: 0.012 to 1.057; df = 22), or gastropods (*d*_native_ = 0.781; CI: 0.623 to 0.940; df = 120), (*d*_non‐native_ = 0.168; CI: −0.037 to 0.373; df = 58) independently.

In order to control for publication bias, where positive results are published more frequently than negative results (i.e., ‘the file‐drawer effect’ (Rosenthal 1984)), we used a fail‐safe sample size. This test measures how many studies with zero effect are required to reject the specified hypothesis (Rosenthal 1979). The fail‐safe test gave a quantity of 37,955 studies (Rosenthal's method), which suggests that publication bias is very unlikely to explain the observed result.

**Figure 2:**
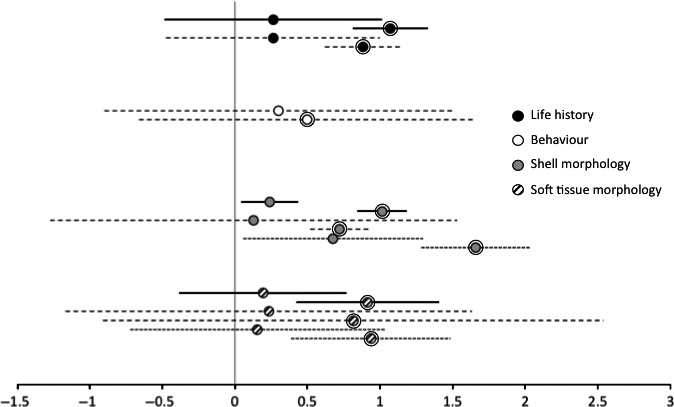
Mean effect sizes (Hedge's *d*) and 95% confidence intervals for the magnitude of phenotypic plasticity. Circle colors designate the four different variables assessed between native predator−prey interactions (enclosed) and interactions between predator and naive prey. Solid lines illustrate both taxa, dashed lines represent gastropod species, while dotted lines represent bivalve species.

For the four different variables in the split data set, not all variables were represented in the literature for the two taxa. This was particularly true for bivalves (Fig. 2). Although life‐history data were available for both taxa, there were only enough data available to perform a meta‐analysis on gastropods. When both taxa were included, the non‐native interaction demonstrated lower effect sizes (*d*_non‐native_ = 0.266; CI: −0.484 to 1.017; df = 7) compared to the native interactions (*d*_native_ = 1.072; CI: 0.813 to 1.331; df = 60). The same pattern prevailed for gastropods (*d*_non‐native_ = 0.263; CI: −0.472 to 0.998; df = 7), (*d*_native_ = 0.885; CI: 0.620 to 1.151; df = 49).

We could only find data about behavioral plasticity for gastropods; where the native group had the largest effect sizes (*d*_non‐native_ = 0.299; CI: −0.900 to 1.498; df = 5), (*d*_native_ = 0.499; CI: −0.662 to 1.660; df = 7). However, this result is not significant since confidence intervals overlap between groups, and with zero.

Furthermore, sufficient data were available for both taxa regarding shell morphology. When both taxa were considered together, native species showed significantly larger magnitudes of phenotypic plasticity, and both groups illustrated low variation (*d*_non‐native_ = 0.240; CI: 0.041 to 0.438; df = 57), (*d*_native_ = 1.014; CI: 0.843 to 1.185; df = 101). However, when we split the data set to each taxon alone, we lost precision with the lower number of studies and the confidence intervals increased. Still, native gastropod interactions (*d*_native_ = 0.721; CI: 0.518 to 0.924; df = 55) and to some extent, native bivalves maintain low confidence intervals (*d*_native_ = 1.660; CI: 1.283 to 2.037; df = 45). On the other hand, this was not true for the non‐native groups of gastropods (*d*_non‐native_ = 0.129; CI: −0.078 to 0.337; df = 40), and bivalves (*d*_non‐native_ = 0.678; CI: 0.060 to 1.297; df = 16). Notably, native bivalves show especially high effect sizes for shell morphology (Fig. 2).

For the final variable tested in isolation, we considered data for phenotypic plasticity on soft tissue morphology equally for gastropods and bivalves. We obtained the same patterns as before, although no significant differences were found between native and non‐native interactions due to large confidence intervals. When both taxa were included, (*d*_non‐native_ = 0.193; CI: −0.383 to 0.769; df = 9), (*d*_native_ = 0.915; CI: 0.422 to 1.408; df = 18), only the native group presented significant phenotypic plasticity. Gastropods demonstrated large confidence intervals for both non‐native (d_non‐native_ = 0.233; CI: −1.169 to 1.635; df = 3) and native interactions (d_native_ = 0.815; CI: −0.909 to 2.539; df = 4). Native bivalves however showed a significant amount of plasticity (d_native_ = 0.937; CI: 0.386 to 1.487; df = 13) while non‐native gastropods did not (d_non‐native_ = 0.155; CI: −0.723 to 1.033; df = 5).

## Discussion

A major question in ecology and evolutionary biology is how native taxa interact with novel species. It is valuable for native prey in a community to recognize invaders at an early stage of the invasion process to be capable of responding appropriately (e.g., producing appropriate defensive traits) and avoiding local extinction (Ogutuohwayo 1990; Knapp 2005; Knapp et al. 2005). For these reasons, we believe a comprehensive approach to estimating the magnitude of plastic responses by native prey in response to novel predators is needed. Such results also need to be evaluated in comparison with background magnitude of plastic responses in native predator−prey interactions.

Here, we have combined a large number of studies from the marine bivalve and gastropod literature and have contrasted the magnitude of plastic defensive responses between prey species exposed to native or non‐native predators.

Surprisingly, we only found three predators representing the non‐native predator species group, and the most common non‐native predator used in these types of studies was the green crab, *Carcinus meanas,* representing 81% of all experiments. Nevertheless, the fact that native prey responses to green crabs were consistently low or absent across multiple prey species and multiple geographical regions enhances the generality of our results. Future studies examining coevolution in the context of predator‐induced plasticity and range expansion (migration of predators or prey), studies on one or a few common non‐native predator species with widespread geographic distribution should therefore provide valuable insight into the dynamics of predator−prey coevolution. When both predator and prey were under consideration, we found that prey species that were exposed to a novel predator, can and do exhibit significant phenotypic plasticity in defensive traits, as their confidence interval did not overlap with zero. However, prey species that had a shared evolutionary history and had coevolved with a native predator, demonstrated significantly higher phenotypic plasticity that was significantly distinct from the predator‐naive group (Fig. 1). Although coevolved prey showed significantly larger effect sizes compared to naive prey when the data were analyzed separately for bivalves and gastropods, the two taxa differed to some extent. Bivalves, regardless if they were exposed to native or novel predators, responded significantly to predator cues. In contrast, whereas gastropods responded significantly to native predators, those exposed to novel predators showed no significant plastic defensive responses. Furthermore, bivalves exhibited larger effect sizes overall compared to gastropods (Fig. 1).

Overall, our results are intuitive. For both bivalves and gastropods, and for all variables measured, interactions between native prey and native predators demonstrate larger phenotypic responses. Bivalves, which are sessile and therefore less mobile than gastropods would reap larger adaptive benefits from plastic morphological responses and so larger plastic responses would be expected; and this is exactly what we find. Further, both gastropods and bivalves have evolved metabolically and structurally expensive shells, these shells can be grown and developed in a very plastic way and shells are often amplified at the expense of soft tissue growth and development when these animals need to increase protection against predators.

In situations where native prey and invasive predators lack a shared evolutionary history, invasive species may have a strong impact on the dynamics of communities and ecosystems (Sergio et al. 2006; Salo et al. 2007). Specifically, predators may have large consumptive effects on prey that lack the appropriate defensive responses to the predator. Invasive predators should therefore incur a larger consumptive effect on prey, in proportion to the prey population's naïveté to the predator (e.g., the prey naïvete hypothesis cf. Cox and Lima (2006)). The degree of naïveté exhibited by the prey (e.g., bivalves and gastropods) may in turn depend on its evolutionary history with the invasive predator or its more general history of undergoing strong predation pressure.

Alternatively, native prey may exhibit naïveté toward invading predators if the invaders show very little similarity with native predators that the native prey experience (Saul and Jeschke 2015). Lack of similarity between invasive predator and native predators could therefore explain, in part, our results. Due to the limited number of studies that include non‐native predators, we cannot use our data set to directly address this possibility; however, the case studies that are available suggest this is unlikely. For example, gastropods in different regions invaded by green crabs exhibit the full spectrum of plastic responses to this non‐native predator; from lack of adaptive responses (Edgell and Neufeld 2008), to specific evolved responses (Edgell 2010; Trussell and Nicklin 2002), and “coincidentally pre‐adapted” responses due to similarity between native and non‐native crabs (Freeman et al. 2013). Indeed, molluscs are often capable of distinguishing between predatory and non‐predatory species (Marko and Palmer 1991) and native and non‐native predators (Edgell and Neufeld 2008; Freeman and Hamer 2009), suggesting that their responses can be highly specific and not reliant on cue similarity.

Finally, if non‐native predators are less effective at consuming novel prey, then prey may respond less strongly due to either a lower actual risk of predation or a lower concentration of chemical risk cues. Thus, one potential alternative explanation for our results could be that prey respond more strongly to native predators because native predators consume more conspecific prey. Currently, we do not have the data to test this possibility. The majority of the analyzed studies included experimental treatments in which predators were fed conspecific prey, but rarely did studies report the actual amount of prey consumed; precluding the possibility of testing the hypothesis that native predators consumed more conspecific prey than non‐native predators.

Our study shows that plastic inducible defenses against novel predators are generally weak or absent. Nevertheless, this is not true for all naïive prey and certain studies reveal intraspecific variation in plasticity against novel predators across populations (Sih et al. 2010; Freeman et al. 2014). Such variation for plastic responses within species may play a crucial role in the evolution of adaptive responses of native species, and the ecological outcomes of novel interactions. Additional information about the fate of native prey populations exposed to novel interactions will be key to understanding how prey populations, taxa, and species with different amount of plasticity will evolve in response to selective pressure from invasive predators. Predicting the evolution of adaptive responses in naive predator−prey interactions will be a challenge however, given that phenotypic plasticity can inhibit species evolvability (Berthon 2015) or place the population on a new adaptive peak from which natural selection can advance (Pfennig et al. 2010; Moczek et al. 2011). Future research should therefore identify whether inducible defenses against non‐native predators are consistent within and across taxa, in order to better understand and predict the effects of invasive predators and employ more effective management strategies.

## Data Archiving

Literature databases corresponding to the systematic review and meta‐analysis will be archived in the Dryad repository.

## Conflict of Interest

The authors declare no conflicts of interest.
